# Obliteration of the Cavity of the Tunica Vaginalis Testis

**Published:** 1844-05-18

**Authors:** 


### OBLITERATION OF THE CAVITY OF THE TUNICA VAGINALIS TESTIS.

Dr. Knox, in his contributions to Anatomy and Physiology, states that during the last eight or ten years he has seen in his dissecting rooms four or five cases of obliteration of the cavity of the tunica vaginalis testis, unconnected, as far as he could ascertain, with any operation or accident. The cavity was entirely obliterated, there not being any other morbid appearance; near the central portion, the tunic presented the characters of common cellular tissue. Dr. Knox adds that he agrees with Portal in believing that adhesions of serous membranes, so extensive as to obliterate their cavity, may take place without inflammation; this he has seen occur twice with reference to the pericardium.—*Ib.*

